# Supplementary figures and images for: Sequence specificity of an essential nuclear localization sequence in Mcm3
Source: PLoS Genet. 2025 Jan 21;21(1):e1011499. doi: 10.1371/journal.pgen.1011499 (PMC11761085; doi:10.1371/journal.pgen.1011499)

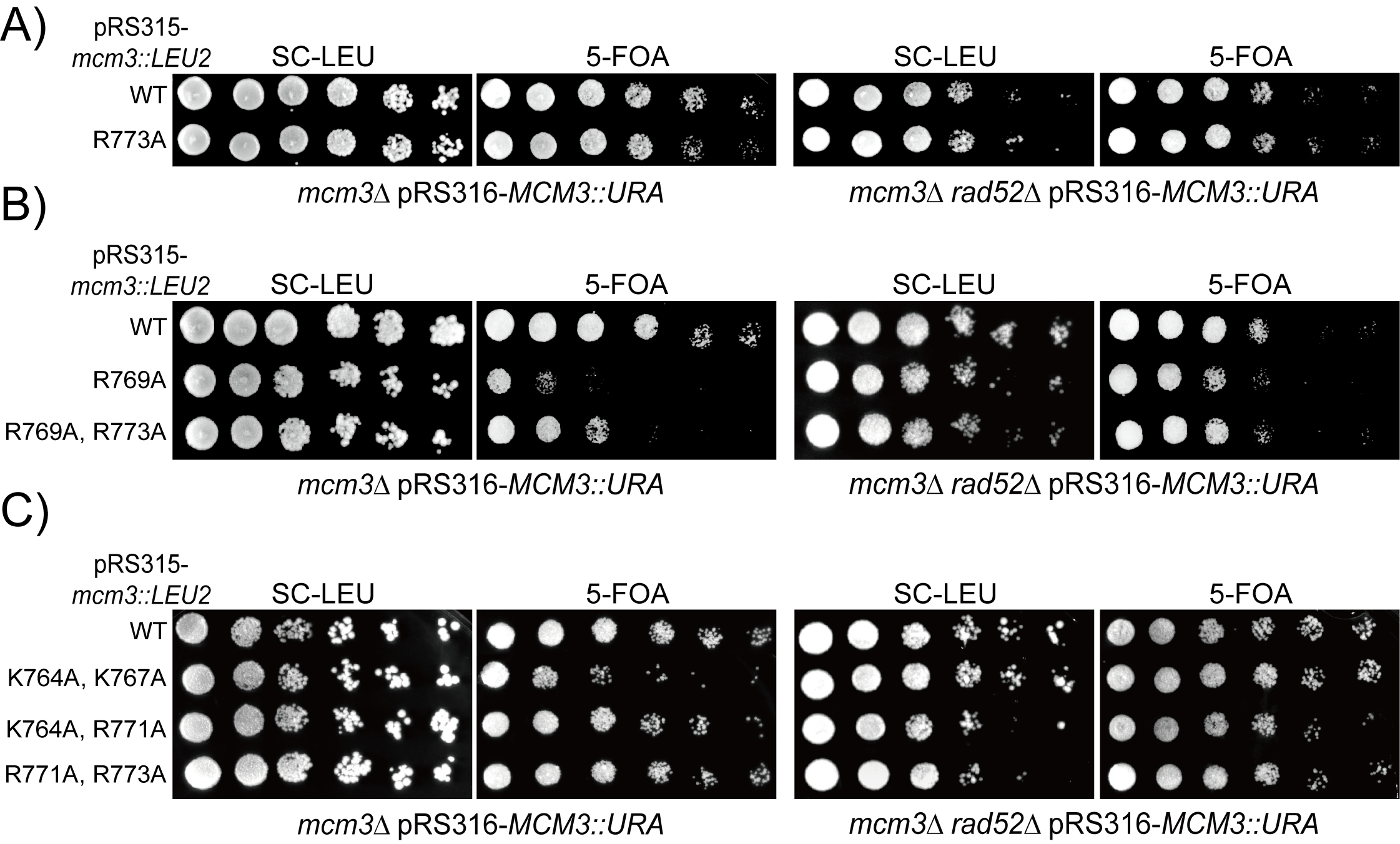

Supplement: S1 Fig — A, B and C) mcm3 mutants with the indicated mutations were analyzed by plasmid shuffling. Mutants were plated on media where the complementing URA3 plasmid was retained (SC-Leu) or lost (5-FOA). (TIF) [file pgen.1011499.s003.tif]

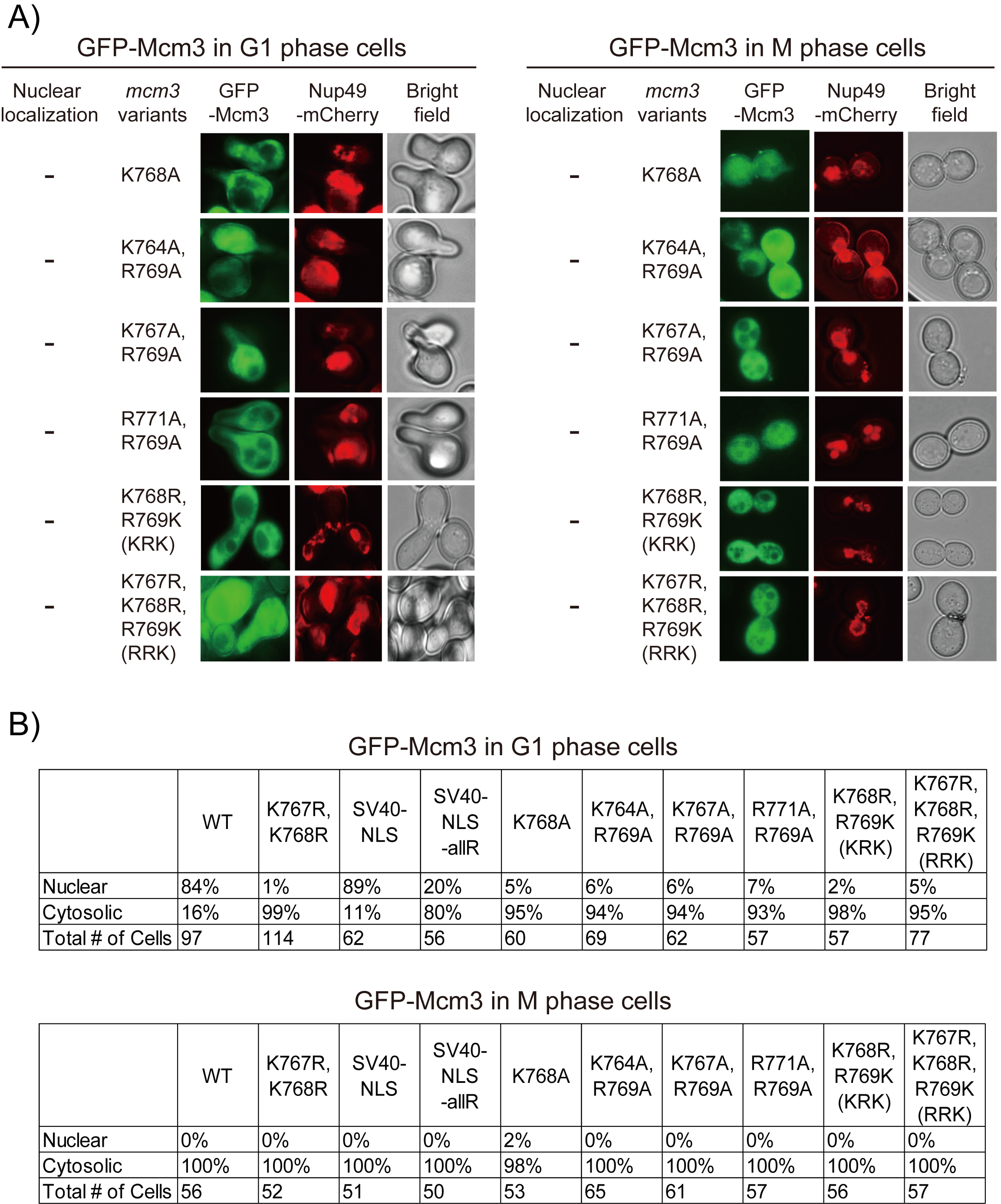

Supplement: S2 Fig — A) Nup49-mCherry cells (HZY1575) transformed with pRS315-GFP-mcm3 plasmids were arrested in G1 or M phase for fluorescence microscopy analysis. Representative images of cells in G1 (left panel) and M (right panel) phases illustrate the localization of GFP-Mcm3 relative to Nup49-mCherry. B) Quantification data for Fig 3B. The number of cells exhibiting either nuclear or cytosolic localization of the indicated GFP-Mcm3 variants, along with the total number of cells counted are indicated in the table. (TIF) [file pgen.1011499.s004.tif]

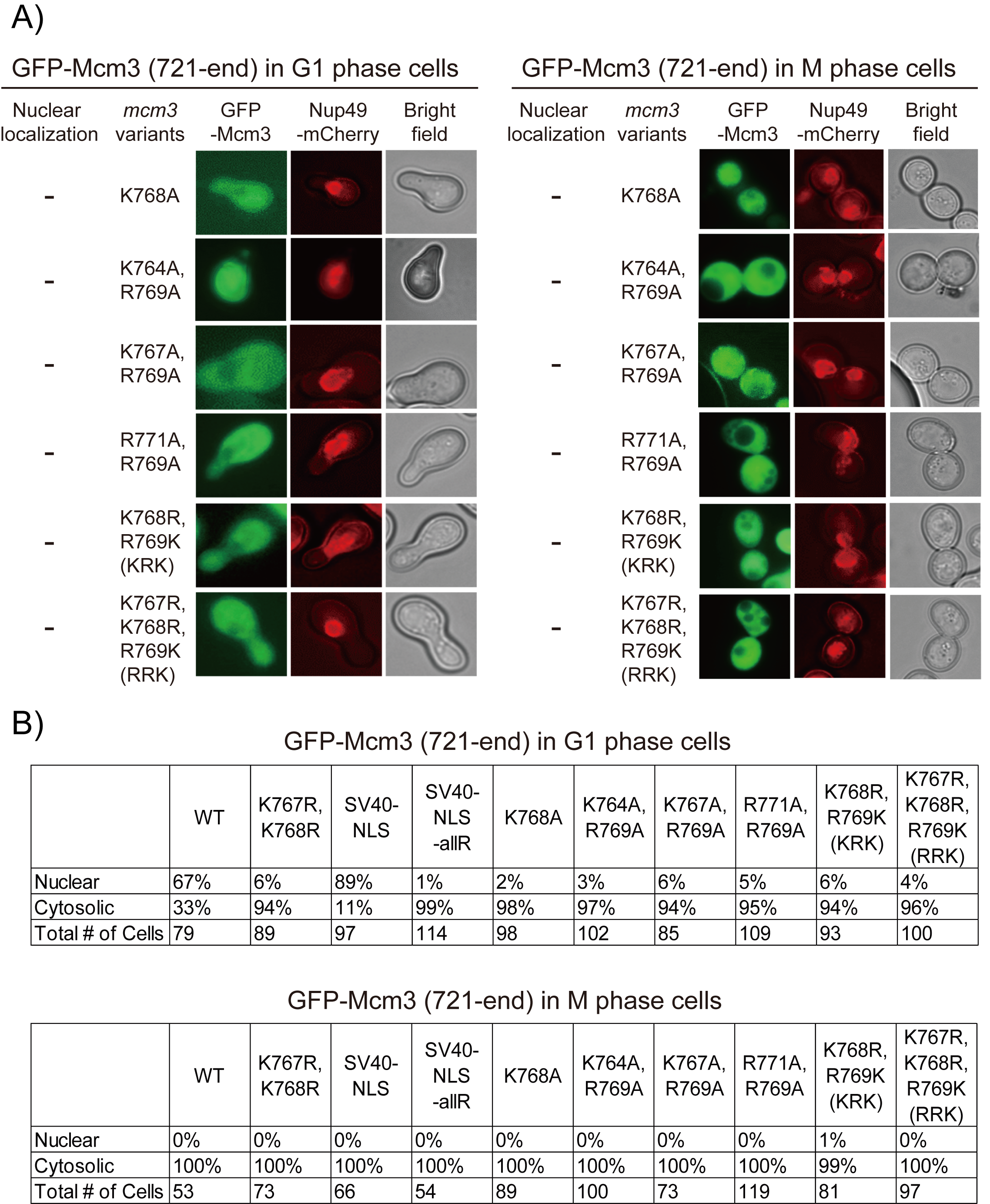

Supplement: S3 Fig — A) Nup49-mCherry cells (HZY1575) transformed with pRS315-GFP-mcm3-721-end plasmids were arrested in G1 or M phase for fluorescence microscopy analysis. Representative images of cells in G1 (left panel) and M (right panel) phases illustrate the localization of GFP-Mcm3 (721-end) relative to Nup49-mCherry. B) Quantification data for Fig 4B. The number of cells exhibiting either nuclear or cytosolic localization of the indicated GFP-Mcm3 (721-end) variants, along with the total number of cells counted are indicated in the table. (TIF) [file pgen.1011499.s005.tif]

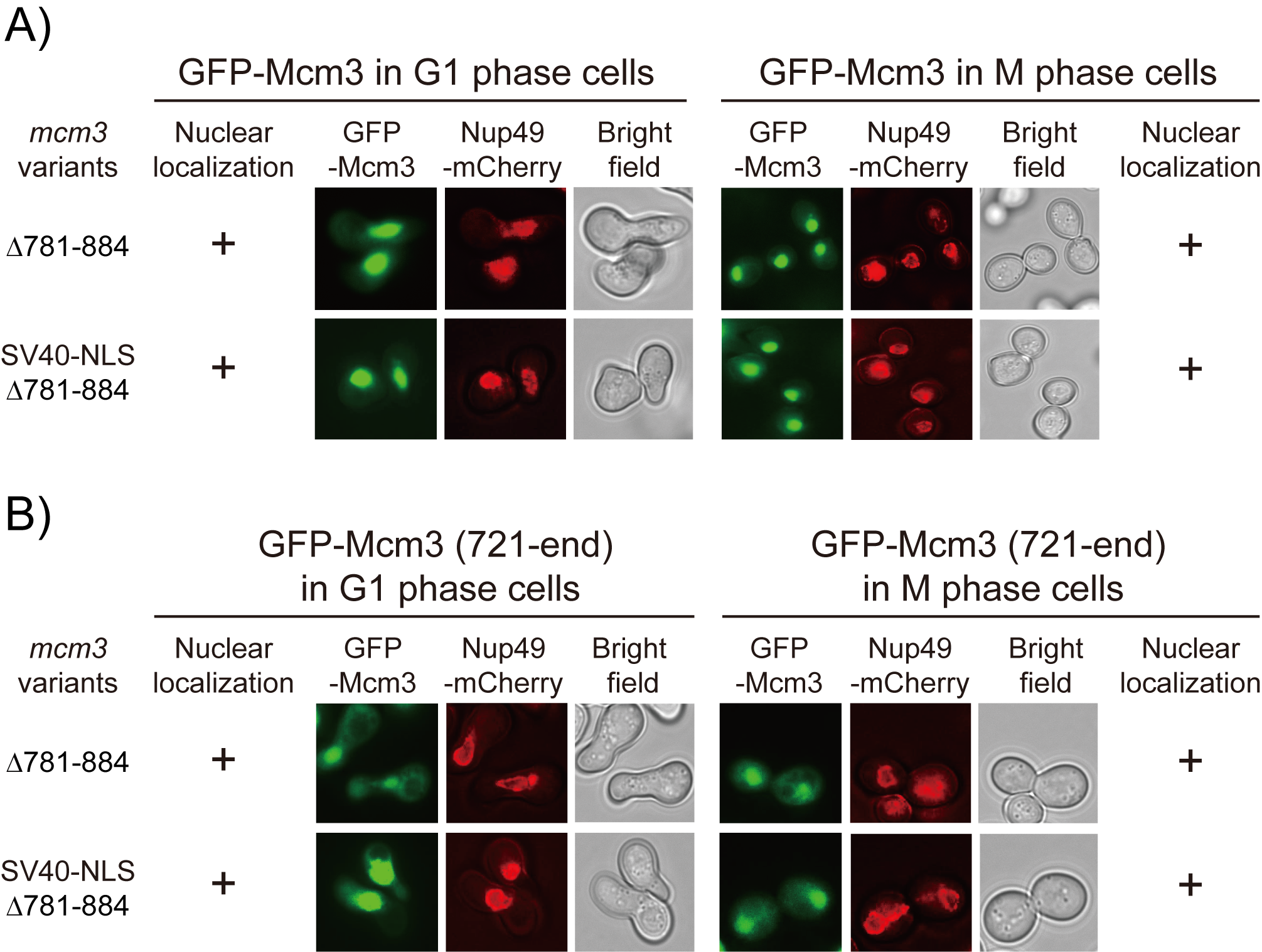

Supplement: S4 Fig — A and B) Nup49-mCherry cells (HZY1575) transformed with pRS315-GFP-mcm3 or pRS315-GFP-mcm3-721-end plasmids with the indicated truncations were arrested in G1 or M phase for fluorescence microscopy analysis. Representative images of cells in G1 (left panel) and M (right panel) phases illustrate the localization of GFP-Mcm3 (S4A Fig) or GFP-Mcm3 (721-end) (S4B Fig) relative to Nup49-mCherry. (TIF) [file pgen.1011499.s006.tif]

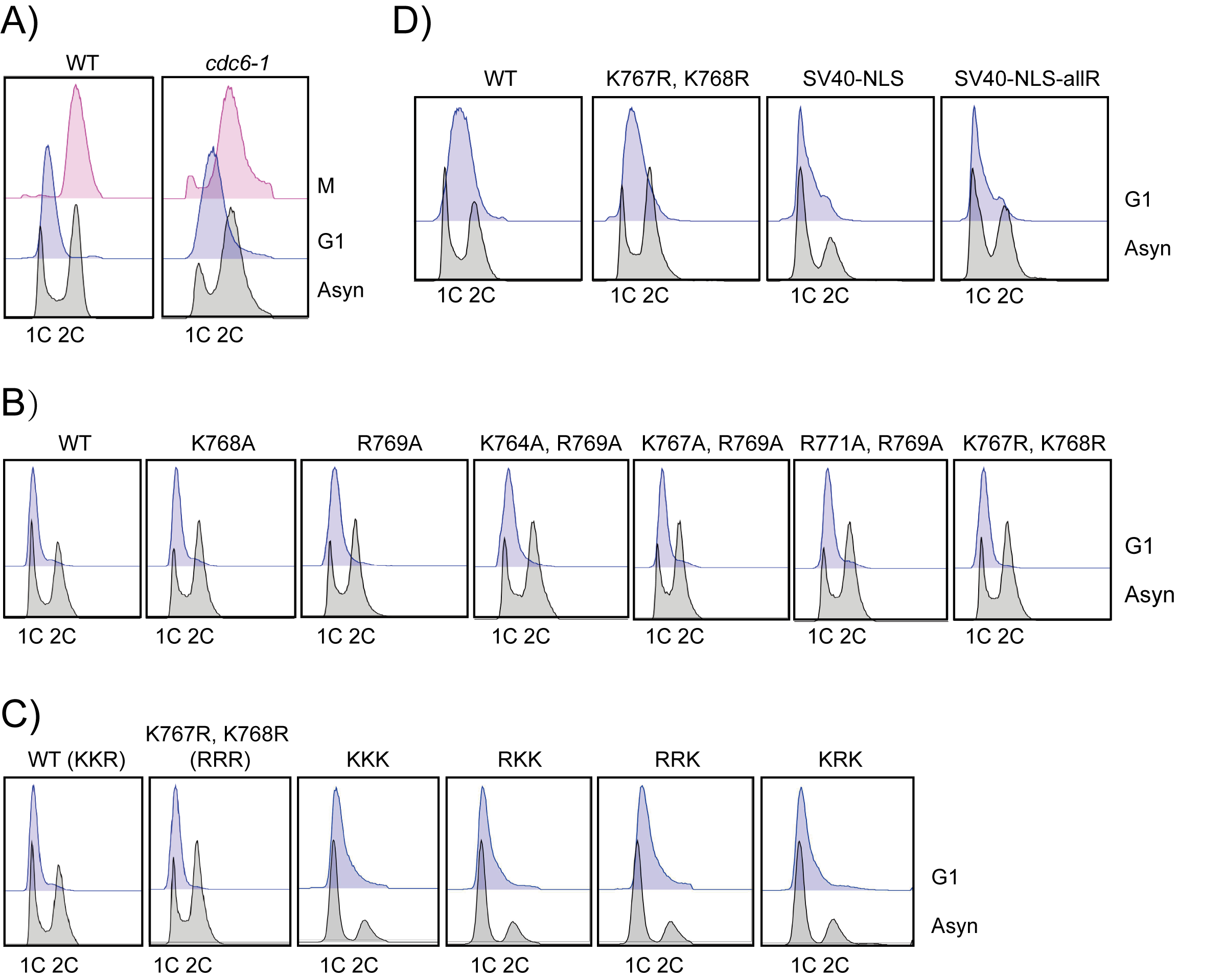

Supplement: S5 Fig — A) Fluorescence-Activated Cell Sorting (FACS) analysis of wild-type (HZY4011) and cdc6-1 (HZY1366) cells following G1 and M phase arrest for Figs 5B and S6C. B) FACS analysis of mcm3 mutants following G1 arrest for Figs 5C, S6D to S6F. C) FACS analysis of mcm3 mutants following G1 arrest for Figs 5D and S6G. D) FACS analysis of mcm3 mutants following G1 arrest for Figs 5D and S6H. (TIF) [file pgen.1011499.s007.tif]

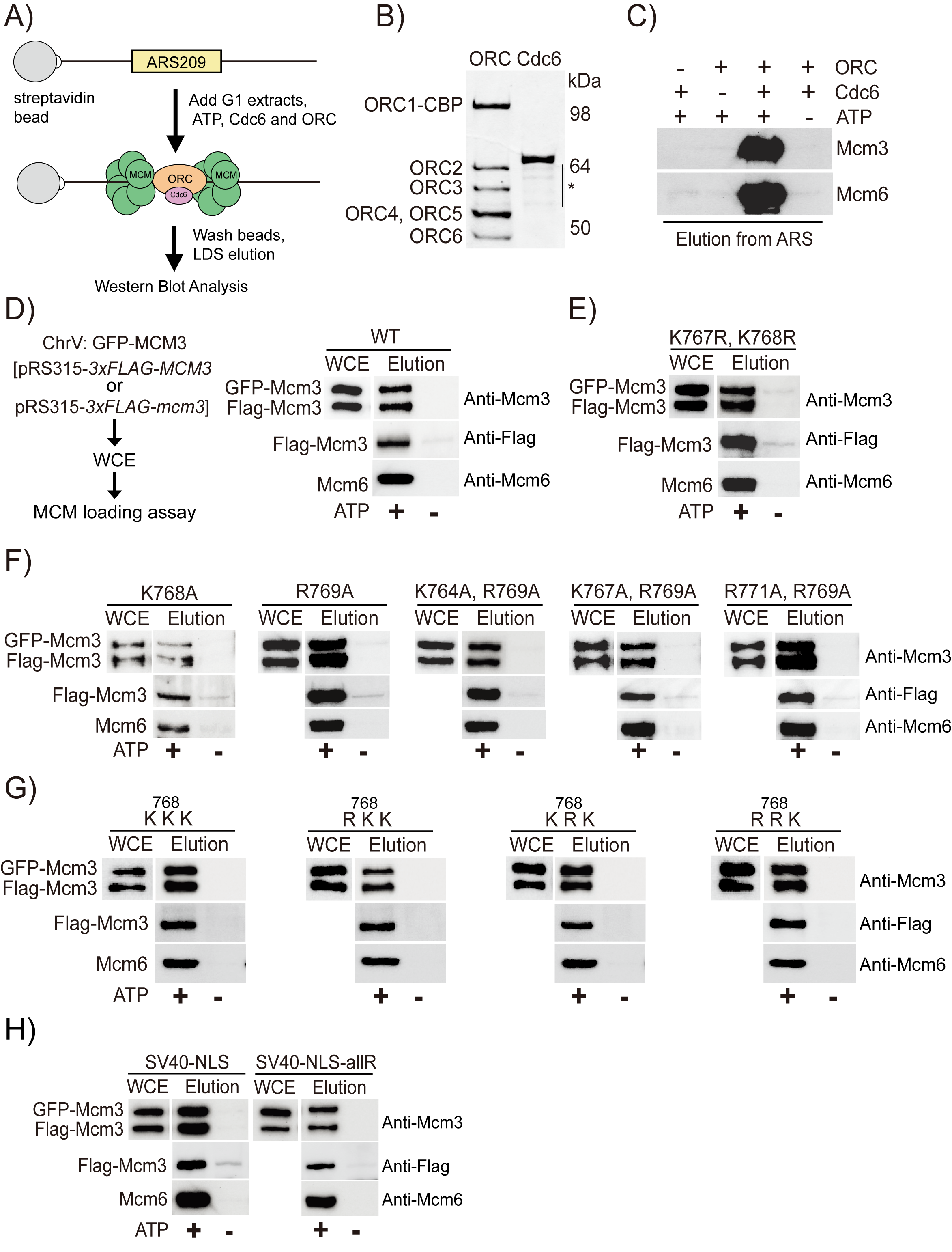

Supplement: S6 Fig — A) A workflow for loading MCM on ARS-containing DNA in vitro. B) Purified ORC and Cdc6 proteins were analyzed by SDS–PAGE and stained with Coomassie blue dye. * Indicates partial degradation of the proteins. C) G1 phase whole cell extracts (WCE) of wild-type (HZY4011) cells were subjected to in vitro MCM loading assay under different conditions: with or without ATP, Cdc6, or ORC. ARS DNA-bound materials were eluted by SDS and analyzed by Western blotting with anti-Mcm3 and anti-Mcm6 antibodies. D to H) GFP-Mcm3 cells (HZY3037) transformed with pRS315-3xFLAG-mcm3 plasmids were arrested in the G1 phase, and the corresponding whole cell extracts (WCE) were subjected to in vitro MCM loading assay. ARS DNA-bound materials were eluted by LDS and analyzed by Western blotting with anti-Mcm3 and anti-Flag antibodies. (TIF) [file pgen.1011499.s008.tif]

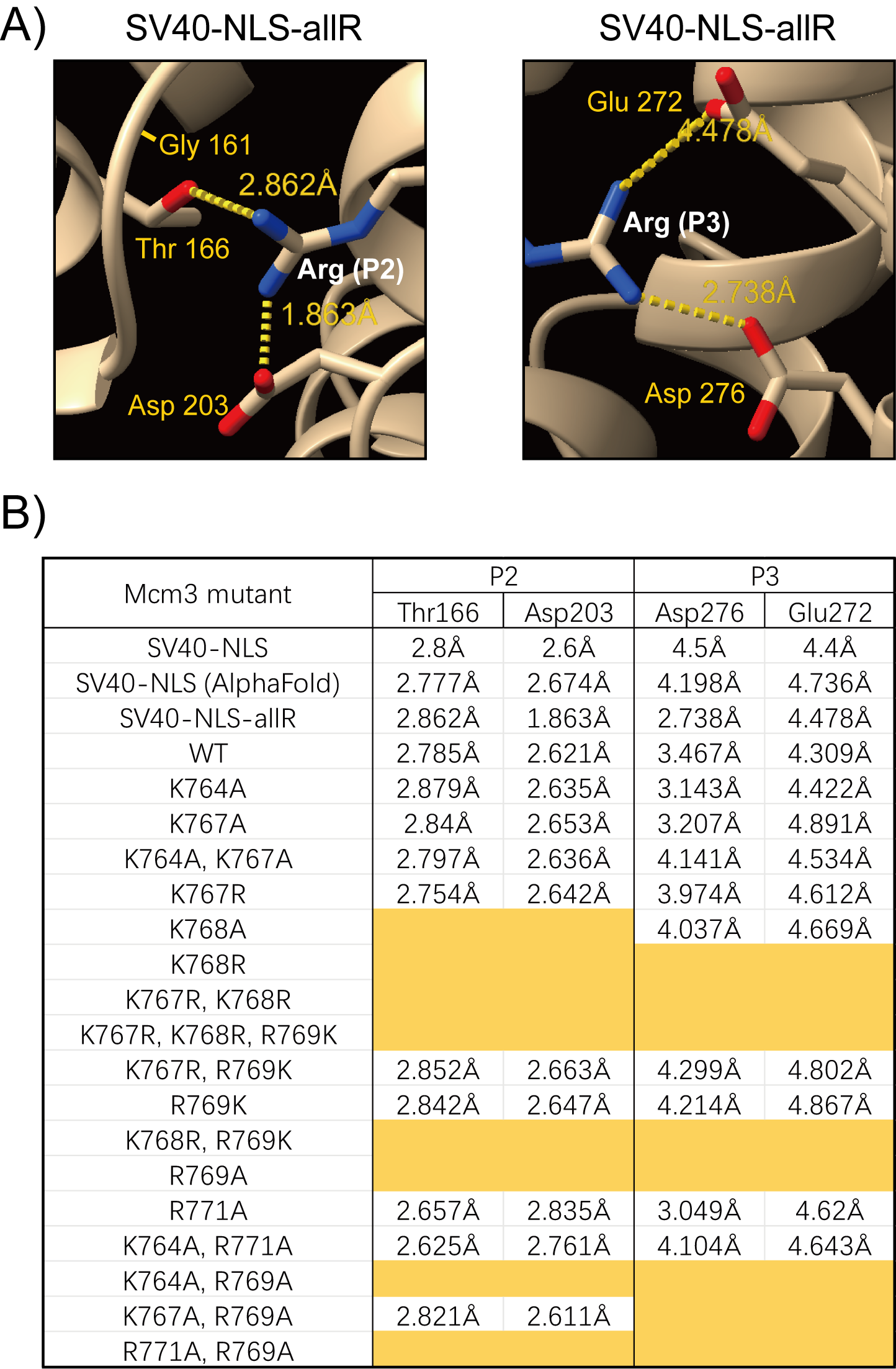

Supplement: S7 Fig — A) AlphaFold predicted interaction between the basic residue at P2 (left panel) or P3 (right panel) in Mcm3SV40-NLS-allR (764KSPRRRRRV772) and Kap60. B) The distances between residues at P2 or P3 within Mcm3’s NLS and key residues in Kap60 were modeled by AlphaFold. Orange indicates that, according to AlphaFold 3 modeling, no specific contact was detected that resembled the published structure of SV40-NLS/karyopherin alpha complex. (TIF) [file pgen.1011499.s009.tif]
