# Supplementary material for: Sequence specificity of an essential nuclear localization sequence in Mcm3
Source: PLoS Genet. 2025 Jan 21;21(1):e1011499. doi: 10.1371/journal.pgen.1011499 (PMC11761085; doi:10.1371/journal.pgen.1011499)
Supplement: Attachment — Submitted filename: Response to PloS genetics Review.docx [file pgen.1011499.s010.docx]

**Dear Editors,**

**First, we would like to thank both reviewers for their helpful and constructive reviews of our manuscript. Our responses to specific points raised by the reviewers are highlighted below:**

Reviewer #1: The cellular localization of yeast MCM helicase is cell-cycle controlled, and it localizes in the nucleus and cytoplasm in the G1 and G2-M phases, respectively (Labib et al. NCB, 1999; Niguen et al. Curr Biol., 2000). MCM comprises six subunits, two of which (MCM2 and MCM3) contain a nuclear localization signal (NLS) (Young et al., Gene Cells, 1997; Liku et al. MBC, 2005).

Taking these previous studies, Wang et al. examined the sequence specificity of NLS in yeast MCM3. The authors tested various mutants having an alanine substitution(s) within the MCM3 NLS and investigated the cell viability (Figs. 1 and 2) and the cellular of the mutant proteins (Figs. 3 and 4). The authors revealed that K768 is the most critical, and R769 plays a secondary role. The authors further confirmed these findings by looking at the chromosome association of the MCM3 mutants (Fig. 5). Finally, the authors used AlfaFold3 to investigate the interaction between the MCM3 mutants and Kap60 and discovered that the basic residue at P2 and P2 positions (MCM3 K768 and R769, respectively) interacts with the Kap60 binding pocket in a manner similar to that observed in SV40 NLS.

All experiments were conducted carefully, and the presented results are convincing. I do not find a significant problem in the manuscript. However, the authors should consider whether the mutations, particularly MCM3 K768A, will not affect the formation of the MCM2-7 complex. If the complex formation was compromised, the mutant cells would be inviable, and the mutant protein might stay in the cytoplasm.

>Regarding whether point mutations in Mcm3 NLS could affect the MCM complex formation, we went one step further: using cell extracts containing various Mcm3 NLS mutants, we performed *in vitro* MCM loading assay and showed that these *mcm3* mutants had no detectable effect on the loading of MCM complexes onto ARS-containing DNA substrate (See Supplementary Figure 6 and Lines 241-249, referring to the unmarked version). This finding strongly suggested that the formation of the MCM complex and its loading onto the ARS-DNA were unaffected by these mutations. This is not surprising considering that these mutations occur in an unstructured region of Mcm3.

Additionally, the authors should be able to show the location of MCM3 NLS within the MCM2-7 complex (related to Fig. 1A). I am asking this because previous publications suggested that MCM2-7 is likely imported and exported to the nucleus as the MCM 2-7 complex (e.g. Liku et al. MBC, 2005).

>Due to its flexible nature, this NLS-containing region of Mcm3 was not detected in the published structures of MCM hexamers (Li et al. Nature, 2015), see Lines 96-97 in the unmarked version. Moreover, our finding showed that truncations of both N-terminal and C-terminal flanking sequences did not affect cell growth (Fig. 1). Finally, GFP fusion of the Mcm3 fragment (amino acid 721 to the end) can localize to the cell nucleus (Figure 4). Together, these findings suggested that the physical location of Mcm3 NLS with respect to the MCM hexamer (the ATPase domain) is not important.

Minor point
Fig. 3B and Fig. 4B: The labelling for "nuclear" and "cytosolic" is opposite.

> Corrected. Thank you!

Reviewer #2: This manuscript provides a comprehensive analysis of the NLS of Mcm3, including analysis of cell viability, nuclear localization of GFP-Mcm3 using fluorescence microscopy, biochemical assays to assess capacity of MCMs to load in vitro, and AF3 predictions. Overall, the manuscript is well written, the results are clear and convincing and make sense. Given the often-ambiguous nature of NLSs, this study provides new insight into what constitutes a monopartite NLS and suggest that AF3 predictions could serve as a useful tool to guide future studies of NLS function by assessing NLS-importin contacts of different mutants. Overall, I think it is suitable for publication, but several minor issues need to be addressed, as detailed below:

Line 91: “structure-guided approach”. The AF3 predictions are in the very end of the paper as the last figure and were not really used for a structure-guided approach. As they are presented, the AF3 predictions provide a potential structural basis for the observed biochemical and genetic results. However, the amount of novel information gained from these models is of limited value, especially given the existing crystal structure of the SV40-NLS with importin alpha. There are no predictions that were generated and tested based on the AF3 data. I would rephrase to say the AF3 predictions could serve as a way to study NLS function, but that this requires testing on other NLSs in future studies and would also point out the limitations.

>We fully agree with this assessment. AF3 was used to rationalize our mutagenesis results, and it did a remarkable job! Our findings suggest that AF3 should be used as a tool to evaluate other NLSs, together with experiments for further validation. We have incorporated this discussion between Lines 304-307 in the unmarked version, pointed out the limitations of AF3 and the requirement for future studies in Lines 343-350 in the unmarked version, and deleted the previous writing at Line 91.

Line 104: Perhaps it is worth explaining the plasmid shuffle concept – the way it’s presented is not clear to those not familiar with classic yeast genetic approaches. E.g. – “Mcm3 is an essential gene and cannot be deleted or mutated. To study mcm3 mutants, we employed a genetic approach that allows us to conditionally remove WT Mcm3 (by selecting against a URA3 plasmid with 5-FOA) while simultaneously complementing with either WT or mutant forms of Mcm3 (selected for with the LEU2 marker).”

>We have included a brief description in the figure legend of Fig. 1B to explain the plasmid shuffling technique.

Line 139: I would add here that this result also fits well with the fact that K768 is highly conserved and is the only invariant K residue across evolution.

>We have incorporated this suggestion at Lines 144-145 in the unmarked version.

Line 188: I would add here – “we considered that mutations introduced into Mcm3 could directly affect MCM loading, leading to the observed defect in cells. To test this, we determined the capacity of these mutants to be loaded on DNA in vitro”. I would add at the end of this paragraph “Therefore, the observed reduction in MCM loading in cells is not because mutants cannot be loaded, but rather because they are not imported into the nucleus”.

>We have incorporated these suggestions at Lines 241-249 in the unmarked version.

Figure 1A: R769 and R771 are also highly conserved and are basic residues in mice/human. These should also be highlighted. However, the only invariant residue is K768.

>We have made the modification in Fig. 1A and its figure legend.

Figure 2C: the annotation of the mutations in the table on the right is incorrect for the two bottom mutants. These should be SV40-NLS and SV40-NLS-allR. The cell growth annotation is also incorrect (should be ++ for SV40-NLS). This table was clearly copied from Figure 2A but not completely edited to reflect the result in 2C…

>We have made the correction, thank you!

Figure 4B: The colors of the graph and the legend seem to have been swapped. The dark part in the graph is clearly nuclear, not cytosolic.

>We have made the correction, thank you!
